# DNA mini‐barcoding of leporids using noninvasive fecal DNA samples and its significance for monitoring an invasive species

**DOI:** 10.1002/ece3.5863

**Published:** 2020-06-05

**Authors:** Nayra T. Rodrigues, Bruno H. Saranholi, Thais A. Angeloni, Nielson Pasqualotto, Adriano G. Chiarello, Pedro M. Galetti Jr

**Affiliations:** ^1^ Departamento de Genética e Evolução Universidade Federal de São Carlos São Carlos Brazil; ^2^ Programa Interunidades de Pós‐Graduação em Ecologia Aplicada (PPGI‐EA) Escola Superior de Agricultura “Luiz de Queiroz” (ESALQ/USP) e Centro de Energia Nuclear na Agricultura (CENA/USP) Universidade de São Paulo Piracicaba Brazil; ^3^ Departamento de Biologia, Faculdade de Filosofia, Ciências e Letras de Ribeirão Preto Universidade de São Paulo Ribeirão Preto São Paulo Brazil

**Keywords:** conservation, European hare, mitochondrial DNA, molecular species identification

## Abstract

Introduced in South America at the end of the 19th century, the European hare population has expanded dramatically and now represents a risk to native Brazilian forest rabbits. Monitoring the invasive *Lepus europaeus* and its coexistence with native *Sylvilagus brasiliensis* is a challenge that can be efficiently addressed by the use of molecular tools. This work describes a set of primers useful for amplifying three mini‐barcodes for the molecular identification of both invasive and native leporid species using degraded fecal DNA. In addition, tests in silico indicate that these mini‐barcodes can successfully amplify the DNA sequences of a number of leporids. These mini‐barcodes constitute a powerful tool for the monitoring and management of the invasive *L. europaeus* and the conservation of native rabbits.

## Graphical abstract

In South America, the European hare is considered an invasive species, representing a risk to native Brazilian forest rabbits. Monitoring the invasive European hare and its coexistence with native forest rabbits is a challenge that can be efficiently addressed by the use of molecular tools. This work describes a set of primers useful for amplifying mini‐barcodes for the molecular identification of both invasive and native leporid species using degraded fecal DNA. These mini‐barcodes constitute a powerful tool for the monitoring and management of the invasive European hare and the conservation of native rabbits.

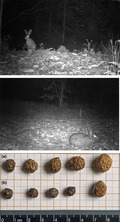

## 1 INTRODUCTION

The introduction of exotic species is a major cause of global biodiversity loss (Bellard, Cassey, & Blackburn, 2016; Mack et al., 2000; McNeely, Mooney, Neville, Schei, & Waage, 2001). The introduction of an alien species into an ecosystem might result in instability, negatively affecting native species through direct or indirect competition, predation, or habitat modification (Blackwell, 2005; Clout & Russell, 2008; Doherty, Glen, Nimmo, Ritchie, & Dickman, 2016; Long, 2003; Mooney et al., 2005).

In South America, the geographic distribution of the introduced European hare (*Lepus europaeus* Pallas, 1778) has been quickly expanding. First introduced in Argentina during 1888 (for a review, see Bonino, Cossíos, & Menegheti (2010)), this exotic leporid has been rapidly spreading through several South American countries, becoming invasive (Bonino et al., 2010; Grigera & Rapoport, 1983; Rosa, Almeida Curi, Puertas, & Passamani, 2017). In Brazil, *L. europaeus* is expanding at a rate of up to 45 km/year (de Faria et al., 2015). No study has thus far investigated in detail the habitat use of this exotic species in Brazil. Although this species can be found in preserved areas (de Faria et al., 2015), its presence seems to be more frequent in areas where native vegetation has been impacted by human activities, such as deforestation and fragmentation, especially in pastures and exotic planting areas (e.g., *Pinus* spp. and *Eucalyptus* spp.) and the agricultural crops that are often found in south and southeastern Brazil (Auricchio & Olmos, 1999).

The native species *Sylvilagus brasiliensis* (Linnaeus, 1758), also known as the Brazilian forest rabbit or tapiti, can be found in the same regions where *L. europaeus* has settled in South America, except for in Chile and Uruguay, where only the alien species is found (Eisenberg & Redford, 1999; Mexican Association for Conservation and Study of Lagomorphs (AMCELA), Romero Malpica & Rangel Cordero, 2008; Bonino et al., 2010). Although *L. europaeus* and *S. brasiliensis* seem to occur most frequently in agricultural crops (Petrovan, Ward, & Wheeler, 2012; Smith, Jennings, & Harris, 2005) and closed vegetation types (Eisenberg & Redford, 1999; Vaughan, Ryan, & Czaplewski, 2011), respectively, research has shown that *S. brasiliensis* is a border species rather than a forest dweller (De Sousa e Silva Júnior, Oliveira, Dias, & Gomes de Oliveira, 2005). In fact, *L. europaeus* and *S. brasiliensis* co‐occur in the contact zones between these habitat types (Salvador & Chiarello, 2016). The potential for competition between the two species by interference and/or exploitation is therefore high. In addition, *L. europaeus* can be a vector of diseases, placing the native species at risk (Cuervo et al., 2015; Edwards, Fletcher, & Berny, 2000). Therefore, correctly assessing the distribution of *L. europaeus* and the areas where this distribution overlaps with the native *S. brasiliensis* habitat is essential for management and conservation measures.

However, the elusive behavior and nocturnal habit of these two species (Reis, Peracchi, Pedro, & Lima, 2011) can represent challenges for data collection based on direct observations, the capture of individuals or even camera traps (Larrucea & Brussard, 2009; Lee, Larsen, Flinders, & Eggett, 2010; Sanchez, Rachlow, Robinson, & Johnson, 2009). In this sense, the search for traces left by animals, such as feces, can be an alternative method for detecting the presence of these species (Davison, Birks, Brookes, Braithwaite, & Messenger, 2002; Laguardia, Jun, Fang‐Lei, Kun, & Riordan, 2015; Souza et al., 2017). However, it is challenging to correctly ascertain the species from which a given fecal sample was derived based solely on morphological aspects due to similarities in the size and shape of feces from different leporid species (Larrucea & Brussard, 2008; Sullivan et al., 2019; Zahratka & Buskirk, 2007).

The use of molecular techniques can be an effective tool for the correct identification of samples, which explains why this approach is increasingly used in research on the wild populations of several taxa (Goossens & Bruford, 2009; Rehnus & Bollmann, 2016; Schwartz, Luikart, & Waples, 2007; Waits & Paetkau, 2005). This approach has also allowed the monitoring of invasive species, even rare invasive species, generating useful information for their management and the conservation of native species (Berry, Sarre, Farrington, & Aitken, 2007; Kovach, Litvaitis, & Litvaitis, 2003).

On the other hand, fecal DNA exposed to environmental conditions can be degraded, limiting the effectiveness of some molecular techniques. Hot weather and humidity, usual in the neotropical regions, accelerate DNA degradation compared with cold and dry conditions of temperate areas (Adams, Goldberg, Bosworth, Rachlow, & Waits, 2011; DeMay et al., 2013; Murphy, Kendall, Robinson, & Waits, 2007; Piggott, 2004). Moreover, sample age also affects DNA degradation (Broquet, Ménard, & Petit, 2007; DeMay et al., 2013). Thus, in neotropics, the use of small sequences or mini‐barcodes for DNA amplification based on fecal samples can improve success.

In this study, we describe three mitochondrial DNA‐based mini‐barcodes (16S rRNA, Cytochrome *b* and Cytochrome Oxidase I) to enable and optimize species identification from the feces of *S. brasiliensis* and *L. europaeus*. The mini‐barcode primers were developed to amplify small fragments of mtDNA (100–200 bp) to optimize DNA amplification and species identification from feces.

## 2 MATERIALS AND METHODS

### 2.1 Development of the mini‐barcode primers

Primer pairs were designed to amplify mini‐barcode sequences from three mitochondrial DNA genes, namely, 16S rRNA, cytochrome b (Cyt*b*), and cytochrome oxidase I (COI; Table 1), using sequences available from GenBank (Benson et al., 2014) for leporids (Appendix S1), in order to evaluate their capability for identification of both focal species, *L. europaeus* and *S. brasiliensis*.

**Table 1:** Primer pair sequences for three mitochondrial genes

mtDNA region	Primer name	Sequences (5′–3′)	Ta (°C)	Amplicon (bp)
COI	COI_Lag	F: CTAATGATTGGAGCCCCTGA R: CCTGCGCCAGCTTCTACTAT	58	116
Cyt*b*	Cyt*b*_Lag	F: ATATCCAAACAACGCAGCAT R: AATGGGTGTTCAACTGGTTG	56	116
16S rRNA	16S_Lag	F: AGAAAGCGTTAAAGCTCAAC R: TCCGATCTGATATAAACTTGTGC	55	167

Abbreviations: bp, base pairs; F, forward; R, reverse; Ta, annealing temperature.

The selected sequences were aligned using ClustalW algorithm (Thompson, Higgins, & Gibson, 1994) implemented in the Geneious software (Kearse et al., 2012). The mini‐barcode primers were designed using Primer 3 Plus, which is available at http://primer3plus.com/web_3.0.0/primer3web_input.htm (Rozen & Skaletsky, 2000). The primer pair selection considered both size of the target fragment and its polymorphism. Shorter fragments, to minimize effects of DNA degradation on the amplification success, showing higher polymorphic information among the tested species were preferable. Hairpins, heterodimers, and/or homodimers were analyzed using OligoAnalyzer 3.1 software (Owczarzy et al., 2008). Finally, the Primer‐BLAST tool (Ye et al., 2012), which allows the analysis of nonspecific annealing, was used to check the primer‐target sequence specificity.

### 2.2 In silico PCR amplification

We performed PCR tests in silico (i.e., using computer simulation) to evaluate the wider applicability of the mini‐barcode primers and verify their annealing ability in other species of Leporidae, including species that occur outside South America. Other potential co‐occurring mammal species with fecal morphology similar to leporids and which may occur in the study region (*Cuniculus paca*, *Dasyprocta leporina*, and *Hydrochoerus hydrochaeris*) were also evaluated to verify the primer specificity (Table 2). Because tissue or feces samples were not available, the sequences available on GenBank were used for in silico tests. The in silico tests were performed using the Primer‐Blast tool (Ye et al., 2012) and determined the ability of the primers to anneal to the GenBank sequences (Benson et al., 2014).

**Table 2:** In silico PCR amplification success (+). Sequence not available in GenBank (*). Non‐in silico amplification result (NA)

Species	COI	Cyt*b*	16S rRNA
*Lepus alleni*	(+)	(+)	*
*Lepus americanus*	(+)	(+)	(+)
*Lepus arcticus*	(+)	(+)	(+)
*Lepus brachyurus*	*	(+)	*
*Lepus californicus*	(+)	(+)	(+)
*Lepus callotis*	*	(+)	*
*Lepus capensis*	(+)	(+)	(+)
*Lepus comus*	(+)	(+)	*
*Lepus coreanus*	(+)	(+)	(+)
*Lepus corsicanus*	(+)	(+)	(+)
*Lepus europaeus*	(+)	(+)	(+)
*Lepus flavigularis*	(+)	(+)	*
*Lepus granatensis*	(+)	(+)	(+)
*Lepus hainanus*	(+)	(+)	(+)
*Lepus insularis*	*	(+)	*
*Lepus mandshuricus*	(+)	(+)	*
*Lepus microtis*	(+)	*	*
*Lepus oiostolus*	(+)	(+)	*
*Lepus othus*	(+)	(+)	(+)
*Lepus peguensis*	(+)	(+)	*
*Lepus saxatilis*	*	(+)	*
*Lepus sinensis*	(+)	(+)	(+)
*Lepus tibetanus*	(+)	(+)	(+)
*Lepus timidus*	(+)	(+)	(+)
*Lepus tolai*	(+)	(+)	(+)
*Lepus townsendii*	(+)	(+)	(+)
*Lepus yarkandensis*	(+)	(+)	*
*Oryctolagus cuniculus*	(+)	(+)	(+)
*Sylvilagus audubonii*	(+)	*	*
*Sylvilagus bachmani*	(+)	*	(+)
*Sylvilagus brasiliensis*	(+)	(+)	*
*Sylvilagus floridanus*	(+)	(+)	(+)
*Sylvilagus nuttallii*	*	(+)	*
*Sylvilagus obscurus*	*	(+)	*
*Sylvilagus palustris*	*	(+)	*
*Sylvilagus transitionalis*	*	(+)	*
*Cuniculus paca*	NA	NA	*
*Dasyprocta leporina*	NA	NA	*
*Hydrochoerus hydrochaeris*	NA	NA	NA

### 2.3 Samples analyzed

**Figure 1:**
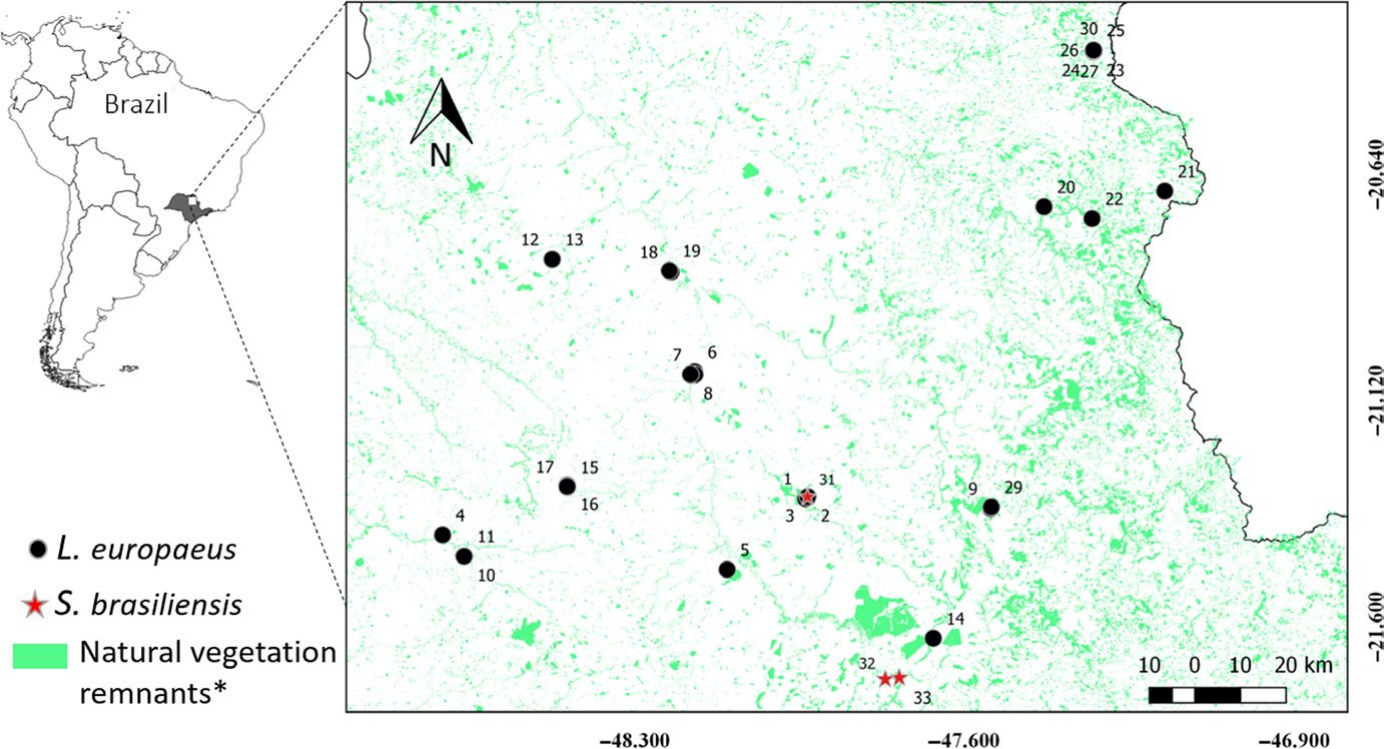
Molecularly identified *Lepus europaeus* and *Sylvilagus brasiliensis* samples collected in the studied area. Numbers represent the sample codes. *Shapefile of “Natural vegetation remnants” obtained from Inventário Florestal da Vegetação Natural do Estado de São Paulo (2010)

A total of 36 potential fecal samples of the native *S. brasiliensis* and invasive *L. europaeus* were collected during a mammal survey carried out in 15 landscapes (200 ha each) during the dry season (April–October) of 2017 and 2018, when the environmental temperature reached up to 35°C. Samples were randomly allocated in an area of approximately 1,500,000 ha in northeastern São Paulo state, Brazil (Figure 1). The sampled landscapes were filtered from an initial pool of 12,000 points in order to ensure spatial independence (i.e., at least 6 km apart between them) and to encompass as much variation as possible in two key variables (percentage of native forest and compositional heterogeneity), following (Pasher et al., 2013), which will be further investigated in a future habitat use study. The time of deposition of the field‐collected pellets was unknown. To optimize and confirm the accuracy of the mini‐barcode primers and perform the PCR tests, control tissue samples obtained from roadkills of both species were used. Both feces and tissue samples were preserved in 95% ethyl alcohol and stored at −20°C, in the biological samples collection of the Laboratory of Molecular Biodiversity and Conservation, Department of Genetics and Evolution, Federal University of São Carlos, Brazil.

DNA from feces was extracted using the QIAamp DNA Stool Mini Kit (Qiagen) following the manufacturer's recommended protocol. We used this specific kit for stool samples to remove the PCR inhibitors. The extraction of DNA from the tissue samples followed the conventional phenol‐chloroform protocol (Sambrook & Russell, 2001).

Sample collection was conducted following the Brazilian legislation, SISBIO (10983‐1), and COTEC (001.155/2017) for sample collection permissions and SISGEN for accessing genetic material authorization (A05D558).

### 2.4 PCR amplification

The PCRs were performed at a 12 μl final volume containing *Taq* Buffer—KCl 1× (Tris–HCl 20 mM pH 8.4 e KCl 50 mM), 3 mM MgCl_2_ (50 mM; Invitrogen) for both COI_Lag and 16S_Lag, 2.5 mM MgCl_2_ for the Cyt*b*_Lag primer pair, 0.2 μM dNTPs, 0.8 pmol of each primer pair, 1U *Taq* DNA Polymerase Platinum (Invitrogen), 50 ng target DNA, and ultrapure water (*q.s.p.*). The PCRs were conducted in a Veriti 96 Well Thermal Cycler (Applied Biosystems). The amplification program consisted of an initial denaturation step at 94°C for 4 min; 35 cycles at 94°C for 30 s, the annealing temperature for each primer (Table 1) for 45 s and 72°C for 45 s; and a final extension step of 12 min at 72°C.

The PCR products were purified using the enzymatic method (ExoSAP‐IT; Affymetrix) and sequenced in both *forward* and *reverse* directions by the 3730xl DNA Analyzer automatic sequencer (Applied Biosystems).

### 2.5 Species identification

The resulting nucleotide sequences for each amplified gene were edited using the Geneious software (Kearse et al., 2012) and compared against the GenBank sequences using the BlastN tool (Madden, 2013) and 97% or higher percentage of identity. For each gene studied, a set of sequences comprised by the reference sequences (Appendix S1), tissue control, and fecal sequences here obtained was aligned using ClustalW algorithm (Thompson et al., 1994) implemented in the Geneious software (Kearse et al., 2012). Genetic distance analyses were conducted using Kimura two‐parameter model (Kimura, 1980), and a neighbor‐joining tree (Saitou & Nei, 1987) was constructed using 1,000 bootstrap replicates.

## 3 RESULTS AND DISCUSSION

All selected mitochondrial regions allowed us to identify the species of leporids tested using both fecal samples and control tissue samples. From the 36 fecal samples analyzed, 30 were identified as *L. europaeus*, three *S. brasiliensis*, and three unidentified, probably due to sample DNA poor quality. Despite the reduced number of *S. brasiliensis* sampled, these results confirm the expectation that the molecular analysis of feces is an efficient tool for the correct identification of Leporidae species and can be used for wild population monitoring (Goossens & Bruford, 2009; Rehnus & Bollmann, 2016; Schwartz et al., 2007; Waits & Paetkau, 2005), particularly the monitoring of invasive species (Berry et al., 2007; Kovach et al., 2003).

The small mini‐barcode sequences obtained (116–167 bp) showed a high number and unmistakable distribution of polymorphic sites (Appendix S2), enabling independently each one for a precise identification of the focal species, and no combination of these mini‐barcodes is mandatory for the species identification success. Thus, any of these mini‐barcodes can be used for molecular identification of *L. europaeus* and *S. brasiliensis*. However, the number of polymorphic sites of 12, 14, and 22 in the COI, Cyt*b*, and 16S rRNA mini‐barcodes between both species (Appendix S3) may suggest the use of 16S rRNA as preferable in case one wants to employ only one of these mini‐barcodes.

The PCR amplification success rates of 90%, 89%, and 83% for 16S_Lag, COI_Lag, and Cyt*b*_Lag, respectively, were comparable among the three mini‐barcodes for the total fecal samples analyzed. Taking in account the environmental condition occurring in the collection sites (temperatures up to 35°C), these can be considered high rates of DNA amplification success, as higher temperatures have a great impact on the DNA degradation (Adams et al., 2011; DeMay et al., 2013; Murphy et al., 2007; Piggott, 2004). High amplification rate (93%) was obtained by Adams et al. (2011) amplifying larger fragments (up to 417 bp) from fecal samples collected during winter, but it was dropped (72%) when fecal samples collected during other seasons were used.

The small size of all three mini‐barcodes here amplified could have favored the success rates obtained (Chaves, Graeff, Lion, Oliveira, & Eizirik, 2012; Rodríguez‐Castro, Saranholi, Bataglia, Blanck, & Galetti, 2018; Walker, Williamson, Sanchez, Sobek, & Chambers, 2016). By targeting small and informative fragments, the mini‐barcode primer pairs described here were able to successfully amplify degraded DNA and produced consistent, positive molecular identification of leporids.

**Figure 2:**
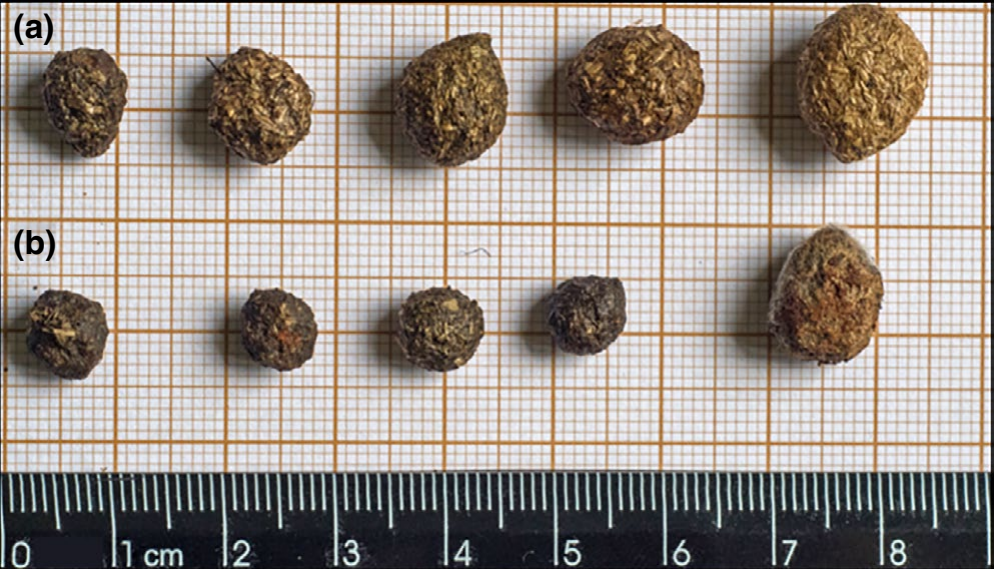
Fecal pellets molecularly identified as from *Lepus europaeus* (a) and *Sylvilagus brasiliensis* (b), highlighting the similarities of size, shape, and color between their feces

Because we were able, with this technique, to identify the invasive *L. europaeus* and native *S. brasiliensis*, which currently co‐occur in several areas throughout South America (Penter, Pedó, Fabián, & Hartz, 2008; Salvador & Chiarello, 2016), these mini‐barcodes have enormous potential as an alternative method for detecting the presence of these species even in low density areas, particularly considering the difficulty of collecting data from these nocturnal and elusive species (Reis et al., 2011) and the fecal morphological similarity observed between both species (Figure 2). Noninvasive genetic sampling and molecular species identification were successfully used for tracking the threatened North American rabbit *Sylvilagus transitionalis* and the presence of two non‐native species, *Sylvilagus floridanus* and *Lepus americanus* (Sullivan et al., 2019), corroborating the use of this approach for monitoring invasive species.

In order to make our study broader, we also evaluated the mini‐barcodes in other leporid species by in silico tests. The in silico PCR amplified the target fragments in all tested species (Table 2), suggesting that the set of mini‐barcode primers developed here has great potential for DNA amplification in other related species and may represent a powerful tool for the establishment of mini‐barcodes, not only for our focal species but also for other leporids. Historical DNA samples from museums and other biological collections, usually having similar highly degraded DNA (Taberlet, Waits, & Luikart, 1999; Wandeler, Hoeck, & Keller, 2007), can also benefit from these tools and extend the use of these mini‐barcodes.

For the three mini‐barcodes obtained, the polymorphism amount was informative for discriminating every leporid species pair tested, except to a few pairwise comparisons (Appendices S2 and S3). All species pair compared could be discriminated through at least one of these min‐barcodes, but *Lepus othus* and *Lepus arcticus* pair, which have been considered highly evolutionary related (Waltari & Cook, 2005; Waltari, Demboski, Klein & Cook, 2004). In addition, the in silico evaluation revealed no potential amplification in the other mammals tested, reinforcing the leporid specificity of these mini‐barcode primers.

In conclusion, since *L. europaeus* population and area of occurrence have significantly increased (Bonino et al., 2010; de Faria et al., 2015; Grigera & Rapoport, 1983), representing a risk of competition and disease for the native *S. brasiliensis* (Cuervo et al., 2015; Edwards et al., 2000), the precise identification of both species by molecular fecal analysis constitutes a powerful tool for effective monitoring changes in occupancy, geographical range, and control of this invasive species.

## CONFLICT OF INTEREST

There are no competing interests to declare.

## AUTHOR CONTRIBUTIONS

AGC, NP, and PMGJ involved in substantial contribution to the conception, design of the work, and data acquisition. NTR, BHS, TAA, and PMGJ involved in contribution in the analysis and interpretation of data. NTR, BHS, NP, AGC, and PMGJ involved in contribution in the writing of the work. NTR, BHS, NP, AGC, and PMGJ involved in contribution in critical review adding intellectual content.

## Data Availability

The GenBank and BOLD Systems accession numbers used to design primers are available in Appendix S1. The obtained mini‐barcode sequences, the polymorphic sites, and the primers organization in each species are available in Appendices S2 and S3. The sequences obtained in this study for *S. brasiliensis* and *L. europaeus* are available on GenBank (Accession numbers: MN633276; MN633277; MN633278; MN633279; MN633280; and MN633281).
