# Supplementary material for: DNA mini‐barcoding of leporids using noninvasive fecal DNA samples and its significance for monitoring an invasive species
Source: Ecol Evol. 2020 Jun 5;10(12):5219–25. doi: 10.1002/ece3.5863 (PMC7319127; doi:10.1002/ece3.5863)
Supplement: Supplementary file 1 [file ECE3-10-5219-s001.docx]

**APPENDIX S1**

GenBank and BOLD Systems accession numbers.

**Table A1.** GenBank and BOLD Systems accession numbers of Leporidae species used for designing primers.

| **mtDNA gene** | **Species** | **Accession number** |
| --- | --- | --- |
| **16S rRNA** | *Lepus americanus* | DQ334833 |
|  | *Lepus arcticus* | DQ334831 |
|  | *Lepus californicus* | DQ334834 |
|  | *Lepus capensis* | GU937113 |
|  | *Lepus coreanus* | NC_024259 |
|  | *Lepus europaeus* | DQ334835 |
|  | *Lepus granatensis* | NC_024042 |
|  | *Lepus hainanus* | JQ219662 |
|  | *Lepus othus* | DQ334830 |
|  | *Lepus sinensis* | NC_025316 |
|  | *Lepus tibetanus* | LC073697 |
|  | *Lepus timidus* | DQ334832 |
|  | *Lepus tolai* | NC_025748 |
|  | *Lepus townsendii* | NC_024041 |
|  | *Oryctolagus cuniculus* | DQ334838 |
|  | *Sylvilagus bachmani* | DQ334837 |
|  | *Sylvilagus floridanus* | DQ334836 |
| **COI** | *Lepus alleni* | KT308120 |
|  | *Lepus americanus* | KT308121 |
|  | *Lepus arcticus* | JF443819 |
|  | *Lepus californicus* | KP735340 |
|  | *Lepus capensis* | HM233194 |
|  | *Lepus comus* | HM233115 |
|  | *Lepus coreanus* | KP993121 |
|  | *Lepus europaeus* | KU250081 |
|  | *Lepus flavigularis* | KT308122 |
|  | *Lepus hainanus* | HG763835 |
|  | *Lepus mandshuricus* | HM233198 |
|  | *Lepus microtis* | KJ192836 |
|  | *Lepus oiostolus* | HM233103 |
|  | *Lepus othus* | KJ397608 |
|  | *Lepus peguensis* | HG763833 |
|  | *Lepus sinensis* | HM233197 |
|  | *Lepus timidus* | KR030070 |
|  | *Lepus tolai* | KX882039 |
|  | *Lepus yarkandensis* | HM233190 |
|  | *Oryctolagus cuniculus* | RDATC030-05* |
|  | *Sylvilagus audubonii* | KU759785 |
|  | *Sylvilagus bachmani* | KU759774 |
|  | *Sylvilagus brasiliensis* | JF444946 |
|  | *Sylvilagus floridanus* | JF443516 |

*sequence available in the BOLD Systems database

| **mtDNA gene** | **Species** | **Accession number** |
| --- | --- | --- |
| **Cyt*b*** | *Lepus alleni* | HQ596458 |
|  | *Lepus americanus* | AY292733 |
|  | *Lepus arcticus* | HQ596461 |
|  | *Lepus brachyurus* | AB058616 |
|  | *Lepus californicus* | AY292731 |
|  | *Lepus callotis* | HQ596469 |
|  | *Lepus capensis* | AJ279411 |
|  | *Lepus comus* | AJ279402 |
|  | *Lepus coreanus* | AB687533 |
|  | *Lepus corsicanus* | AF157464 |
|  | *Lepus europaeus* | HQ596474 |
|  | *Lepus flavigularis* | HQ596475 |
|  | *Lepus granatensis* | HQ596476 |
|  | *Lepus hainanus* | HM232986 |
|  | *Lepus insularis* | HQ596478 |
|  | *Lepus mandshuricus* | HM233091 |
|  | *Lepus oiostolus* | HM233065 |
|  | *Lepus othus* | HQ596479 |
|  | *Lepus peguensis* | HG763845 |
|  | *Lepus saxatilis* | AY292730 |
|  | *Lepus sinensis* | AJ279418 |
|  | *Lepus timidus* | AJ279425 |
|  | *Lepus townsendii* | AY292729 |
|  | *Lepus yarkandensis* | HM233059 |
|  | *Oryctolagus cuniculus* | AJ001588 |
|  | *Sylvilagus audubonii* | AY292722 |
|  | *Sylvilagus brasiliensis* | MH115207 |
|  | *Sylvilagus floridanus* | AY292724 |
|  | *Sylvilagus nuttallii* | AY292723 |
|  | *Sylvilagus obscurus* | AY292725 |
|  | *Sylvilagus palustris* | AY292727 |
|  | *Sylvilagus transitionalis* | AF034256 |
