# Supplementary material for: DNA mini‐barcoding of leporids using noninvasive fecal DNA samples and its significance for monitoring an invasive species
Source: Ecol Evol. 2020 Jun 5;10(12):5219–25. doi: 10.1002/ece3.5863 (PMC7319127; doi:10.1002/ece3.5863)
Supplement: Supplementary file 2 [file ECE3-10-5219-s002.docx]

**APPENDIX S2**

Mini-barcode sequences, polymorphic sites and the primers organization in each species.

**Table A2.** Mini-barcode sequences based on the COI*,* Cyt*b* and 16S rRNA genes of the studied species, highlighting the polymorphic sites. The forward and reverse primers designed in *L. europaeus* and their respective organization in other Leporidae species are indicated in gray.

**COI**

*Lepus europaeus* 1 CTAATGATTGGAGCCCCTGATATAGCTTTCCCCCGAATAAACAATATAAGCTTTTGACTT

*Lepus alleni* 1 .....A............................................T........C

*Lepus americanus* 1 .....A.......................T........G...........T........C

*Lepus arcticus* 1 .....A.......................T.............................C

*Lepus californicus* 1 .....A................................G...........T........C

*Lepus capensis* 1 ....................C........T.............................C

*Lepus comus* 1 T............................T..............C...............

*Lepus coreanus* 1 .....A.......................T.............................C

*Lepus flavigularis* 1 .....A...........C...........T........G...........T.....G..C

*Lepus hainanus* 1 .......................G.....T.......................C......

*Lepus mandshuricus* 1 ....................C........T.............................C

Lepus microtis 1 .....A....................C..........................C......

*Lepus oiostolus* 1 T..........G.................T..............C...............

*Lepus othus* 1  *.....A.......................T.............................C Lepus peguensis* 1 .......................G.....T..A..G........................

*Lepus sinensis* 1 ....................C........T.............................C

*Lepus timidus* 1 .....A.......................T.............................C

Lepus tolai 1 T....A..............C.....A..T........G..T...........C......

*Lepus yarkandensis* 1 .....A..............C.....C..T.............................C

*Oryctolagus cuniculus* 1 ..G..A.....G..T.....C.....C..............T.....G.....C......

*Sylvilagus audubonii* 1 ..T..A..............C........T.......................C......

*Sylvilagus brasiliensis* 1 .....A.......................T..............................

*Sylvilagus floridanus* 1 .....A..............C........T..............C...............

Sylvilagus bachmani 1 .....A.................G..C................................C

*Lepus europaeus* 61 CTCCCACCATCTTTCCTTCTCTTATTAGCCTCATCTATAGTAGAAGCTGGCGCAGG

*Lepus alleni* 61 .....C...............C.............C...........C........

*Lepus americanus* 61 .....C...............C.............C...........C........

*Lepus arcticus* 61 ....................T.............................T.....

*Lepus californicus* 61 .....C...............C.............C...........C........

*Lepus capensis* 61 ........................................................

*Lepus comus* 61 .....C..............T..G.................T..............

*Lepus coreanus* 61 ....................T.............................T.....

*Lepus flavigularis* 61 .....C.............................C....................

*Lepus hainanus* 61 ........................................................

*Lepus mandshuricus* 61 ..T..................C..................................

Lepus microtis 61 ........................C................T..............

*Lepus oiostolus* 61 .....C..............T..G.................T..............

*Lepus othus* 61  *....................T.............................T.....*

*Lepus peguensis* 61 ..................................................T.....

*Lepus sinensis* 61 ........................................................

*Lepus timidus* 61 ....................T....................T........T.....

Lepus tolai 61 ..T..T.....C..T..A..TC..C.T.....G..C..G..G..G..C..A.....

*Lepus yarkandensis* 61 ....................T...................................

*Oryctolagus cuniculus* 61 .....C..T..A........TC..C.......C..A..............G..G..

*Sylvilagus audubonii* 61 ..T........C.....C..TC.CC.T..T.....G..............A.....

*Sylvilagus brasiliensis* 61 ...........C.....C...C..C.T..T..T..A...........C..G.....

*Sylvilagus floridanus* 61 .....T..G..C..T......C..C.T..T.....A...........C..G..G..

Sylvilagus bachmani 61 ........C.....T..C..TC.GC.T........A..............T.....

**Cyt*b***

*Lepus europaeus* 1 ATATCCAAACAACGCAGCATAATATTCCGCCCCATTAGCCAAGTCCTTTTCTGAATCCTT

*Lepus alleni*  1 .............................A.....C........T.....T........C

*Lepus americanus* 1 .............................A..............T..C...........G

*Lepus arcticus* 1 .............................A..G...........T..C...........C

*Lepus brachyurus* 1 ..G..........................A...G.C.......CT..C......G.....

*Lepus californicus* 1 .............................A.....C..T.....T.....T........A

*Lepus callotis* 1 .............................A.....C........T.....T..G.....C

*Lepus capensis* 1 .............................A..............T..C...........C

*Lepus comus* 1 ..........................................ACT..............G

*Lepus coreanus* 1 .............................A..G...........T..C...........C

*Lepus corsicanus* 1 .....................G.......A.................C........T..C

*Lepus flavigularis* 1 .............................A.....C........T.....T........C

*Lepus granatensis* 1 ..............T..............A..T...........T..............C

*Lepus hainanus* 1 .....T.................G.....G........T.....T.....T........C

*Lepus insularis* 1 .............................A.....C........T.....T........A

*Lepus mandshuricus* 1 .............................A....................T.........

*Lepus oiostolus* 1 ..G.........................................T..............C

*Lepus othus* 1 .............................A..G...........T..C...........C

*Lepus peguensis* 1 .....T.................G.....A........T.....T.....T........C

*Lepus saxatilis* 1 .............................A.....C.......................C

*Lepus sinensis* 1 ............T.......G........A..T..........C...............C

*Lepus timidus* 1 .............................A..G...........T..C...........C

*Lepus townsendii* 1 .............................A..............T..C...........C

*Lepus yarkandensis* 1 .............................A..T...........T...........T..C

*Oryctolagus cuniculus* 1 ..G..T........T.....G........A........T........A......G.T..C

*Sylvilagus aquaticus* 1 .YG..T..............G........A.....C..T.....T..M..S...G.....

*Sylvilagus audubonii* 1 .CT.................G........G...........G..T..C.....GG.....

*Sylvilagus brasiliensis* 1 .............................A...T.C......A....C............

*Sylvilagus floridanus* 1 ....................G.....T..A.....C........T..A............

*Sylvilagus nuttallii* 1 .CC...........T..T..G........A.................C......G.G...

*Sylvilagus obscurus* 1 ..C.......................T..G..T..C........T..A......G....A

*Sylvilagus palustris* 1 .................T...........A.....C......A.T..C......G.....

*Sylvilagus transitionalis* 1 ..C.......................T..G..T..C........T..A......G....A

*Lepus europaeus* 61 GTCGCAGACCTTCTTACACTCACATGAATCGGAGGGCAACCAGTTGAGCACCCCTT

*Lepus alleni*  61 ..T..G........C........G.....T.....A..G........A.....A..

*Lepus americanus* 61 ..T...........A...........G........A...........A.....A..

*Lepus arcticus* 61 ..T...........A...........G........A...........A.....A..

*Lepus brachyurus* 61 ..T...........C...........G.....T..............A.....A..

*Lepus californicus* 61 ..T...........C..............T.....A...........A.....A..

*Lepus callotis* 61 ..T...........C.....G........T.....A...........A.....A..

*Lepus capensis* 61 ..T...........G....................A...........A.....A..

*Lepus comus* 61 A.T.......................G..T.....A..............T..A..

*Lepus coreanus* 61 ..T...........A...........G....................A.....A..

*Lepus corsicanus* 61 ..T...........G...........G..T.....A...........A.....A..

*Lepus flavigularis* 61 ..T...........C..............T.....A...........A.....A..

*Lepus granatensis* 61 ..T.................T.....G........A...........A........

*Lepus hainanus* 61 A...................A.....G..T.....C...........A.....A..

*Lepus insularis* 61 ..T...........C..............T.....A...........A.....A..

*Lepus mandshuricus* 61 ..............G..............T.................A.....A..

*Lepus oiostolus* 61 ..T..........................T.....A...........A.....A..

*Lepus othus* 61 ..T...........A...........G........A...........A.....A..

*Lepus peguensis* 61 ....................G........T.................A.....A..

*Lepus saxatilis* 61 ........T.....C.....T..............A.....C..C..A.....A..

*Lepus sinensis* 61 A....T........G....................C...........A.....A..

*Lepus timidus* 61 ..T...........G...........G........A...........A.....A..

*Lepus townsendii* 61 ..T...........A...........G........A...........A.....A..

*Lepus yarkandensis* 61 ..T...........G..............T.....A...........AT....A..

*Oryctolagus cuniculus* 61 ........T.....C....................C........A..A.....G..

*Sylvilagus aquaticus* 61 ...........C..C.................G..C.....C..C...Y....G..

*Sylvilagus audubonii* 61 ..T..T........C...........G..T.....T.....C........T..A..

*Sylvilagus brasiliensis* 61 A.T..T..T.....C..............T.....A...........A.....A..

*Sylvilagus floridanus* 61 ..............C.................G..C..G..C.....A..T..A..

*Sylvilagus nuttallii* 61 .....C..T.....C.................G..C.....T.....A.....A..

*Sylvilagus obscurus* 61 ..T...........C....................A.....C..C..A.....A..

*Sylvilagus palustris* 61 ..T..G.....C..C....................C..G..C.....A.....G..

*Sylvilagus transitionalis* 61 ..T...........C....................A.....C..C..A.....A..

**16S rRNA**

*Lepus europaeus* 1 AGAAAGCGTTAAAGCTCAACAATCAAAAT-CAACTTAATTCCTATAT-TTAA-GAACGAA

*Lepus americanus* 1 .............................-T........C.T..C..-.A..-A......

*Lepus arcticus* 1 .....................G.......-T.............G..-....-.......

*Lepus californicus* 1 .............................CT........C....C..-....-A......

*Lepus capensis* 1 .....................G.......-T........C....G..-....-.......

*Lepus coreanus* 1 .....................G.......-T.............G..-....-.......

*Lepus granatensis* 1 .....................G.......-T.............G..-....-.......

*Lepus hainanus* 1 .............................-.........C.......-....-A......

*Lepus othus* 1 .....................G.......-T.............G..-....-.......

*Lepus sinensis* 1 .............................-T........C....G.A-.C..-A......

*Lepus tibetanus* 1 ............................C-T.............A..-....CA......

*Lepus timidus* 1 .....................G.......-T.............G..-....-A......

*Lepus townsendii* 1 .....................G.......-T........C....G..-....-.......

*Lepus tolai* 1 .....................G.......-T........C....G..-....-.......

*Oryctolagus cuniculus* 1 ......................CT....AC.........AAGA....-..T.-A......

*Sylvilagus bachmani* 1 ............................G-.....C...CAAA.C..-C...TA......

*Sylvilagus brasiliensis* 1 .............................-T........ATTA.A..A..T.CC......

*Sylvilagus floridanus* 1 .............................-T........CAAA....TCA.CCA......

*Lepus europaeus* 58 CTCTTATAATCCCTTAACTGGACTAATCTATAAATTTATAGAAGAAATAATGCTAATATG

*Lepus americanus* 58 ........GA.-.C..............................................

*Lepus arcticus* 58 .........C.-.C....................C.........................

*Lepus californicus* 59 .........CTT.C..............................................

*Lepus capensis* 58 .........C.-.C....................C.........................

*Lepus coreanus* 58 .........C.-.C....................C.........................

*Lepus granatensis* 58 .........C.-.C....................C.........................

*Lepus hainanus* 58 .........A.-.CC.............................................

*Lepus othus* 58 .........C.-.C....................C.........................

*Lepus sinensis* 58 ...........-.C.........C..........CA........................

Lepus tibetanus 59 .........C.-.C....................C.........................

*Lepus timidus* 58 .........C.-.C....................C.........................

*Lepus townsendii* 58 .........C.-.C....................C.........................

Lepus tolai 58 ........GC.-.C....................C.........................

*Oryctolagus cuniculus* 59 .........A.-.C.....................C.......................A

Sylvilagus bachmani 59 ...C.....A.-T..G.......C..........A........................A

*Sylvilagus brasiliensis* 60 ...C.....A.-......................A............C...........A

*Sylvilagus floridanus* 60 ...C.....A.-......................A............C...........A

*Lepus europaeus* 118 AGTAACAAGAATTCTATTCTCCTTGCACAAGCCTATATCAGATCGGA

*Lepus americanus* 117 .............T.................................

*Lepus arcticus* 117 .............TA................................

*Lepus californicus* 119 .............TA................................

*Lepus capensis* 117 ..............A................................

*Lepus coreanus* 117 .............TA................................

*Lepus granatensis* 117 .............T.................................

*Lepus hainanus* 117 .............A.................................

*Lepus othus* 117 .............TA................................

*Lepus sinensis* 117 ............CTA................................

Lepus tibetanus 118 .............T.................................

*Lepus timidus* 117 .............TA................................

*Lepus townsendii* 117 .............TA................................

Lepus tolai 117 ..............A................................

*Oryctolagus cuniculus* 118 ............A...................T..............

Sylvilagus bachmani 118 ...........CAACG...............TT..............

*Sylvilagus brasiliensis* 119 ...........CAA.G...............TT..............

*Sylvilagus floridanus* 119 ...........CCA.G................T..............
