# Supplementary material for: DNA mini‐barcoding of leporids using noninvasive fecal DNA samples and its significance for monitoring an invasive species
Source: Ecol Evol. 2020 Jun 5;10(12):5219–25. doi: 10.1002/ece3.5863 (PMC7319127; doi:10.1002/ece3.5863)
Supplement: Supplementary file 3 [file ECE3-10-5219-s003.docx]

**APPENDIX S3**

Number of polymorphic sites

**Table A3.** Number of polymorphic sites between species pair.

**COI**

|  | A | B | C | D | E | F | G | H | I | J | K | L | M | N | O | P | Q | R | S | T | U | V | W | X |
| --- | --- | --- | --- | --- | --- | --- | --- | --- | --- | --- | --- | --- | --- | --- | --- | --- | --- | --- | --- | --- | --- | --- | --- | --- |
| A. *Lepus europaeus* |  |  |  |  |  |  |  |  |  |  |  |  |  |  |  |  |  |  |  |  |  |  |  |  |
| B. *Lepus alleni* | 7 |  |  |  |  |  |  |  |  |  |  |  |  |  |  |  |  |  |  |  |  |  |  |  |
| C. *Lepus americanus* | 9 | 2 |  |  |  |  |  |  |  |  |  |  |  |  |  |  |  |  |  |  |  |  |  |  |
| D. *Lepus arcticus* | 5 | 8 | 8 |  |  |  |  |  |  |  |  |  |  |  |  |  |  |  |  |  |  |  |  |  |
| E. *Lepus californicus* | 8 | 1 | 1 | 9 |  |  |  |  |  |  |  |  |  |  |  |  |  |  |  |  |  |  |  |  |
| F. *Lepus capensis* | 3 | 8 | 8 | 4 | 9 |  |  |  |  |  |  |  |  |  |  |  |  |  |  |  |  |  |  |  |
| G. *Lepus comus* | 7 | 12 | 12 | 8 | 13 | 8 |  |  |  |  |  |  |  |  |  |  |  |  |  |  |  |  |  |  |
| H. *Lepus coreanus* | 5 | 8 | 8 | 0 | 9 | 4 | 8 |  |  |  |  |  |  |  |  |  |  |  |  |  |  |  |  |  |
| I. *Lepus flavigularis* | 9 | 6 | 4 | 8 | 5 | 8 | 12 | 8 |  |  |  |  |  |  |  |  |  |  |  |  |  |  |  |  |
| J. *Lepus hainanus* | 3 | 10 | 10 | 6 | 11 | 4 | 8 | 6 | 10 |  |  |  |  |  |  |  |  |  |  |  |  |  |  |  |
| K. *Lepus mandshuricus* | 5 | 8 | 8 | 6 | 9 | 2 | 10 | 6 | 10 | 6 |  |  |  |  |  |  |  |  |  |  |  |  |  |  |
| L. *Lepus microtis* | 5 | 10 | 12 | 8 | 11 | 8 | 10 | 8 | 12 | 6 | 10 |  |  |  |  |  |  |  |  |  |  |  |  |  |
| M. *Lepus oiostolus* | 8 | 13 | 13 | 9 | 14 | 9 | 1 | 9 | 13 | 9 | 11 | 11 |  |  |  |  |  |  |  |  |  |  |  |  |
| N. *Lepus othus* | 5 | 8 | 8 | 0 | 9 | 4 | 8 | 0 | 8 | 6 | 6 | 8 | 9 |  |  |  |  |  |  |  |  |  |  |  |
| O. *Lepus peguensis* | 5 | 12 | 12 | 6 | 13 | 6 | 10 | 6 | 12 | 4 | 8 | 10 | 11 | 6 |  |  |  |  |  |  |  |  |  |  |
| P. *Lepus sinensis* | 3 | 8 | 8 | 4 | 9 | 0 | 8 | 4 | 8 | 4 | 2 | 8 | 9 | 4 | 6 |  |  |  |  |  |  |  |  |  |
| Q. *Lepus timidus* | 6 | 9 | 9 | 1 | 10 | 5 | 7 | 1 | 9 | 7 | 7 | 7 | 8 | 1 | 7 | 5 |  |  |  |  |  |  |  |  |
| R. *Lepus tolai* | 24 | 22 | 20 | 22 | 21 | 23 | 23 | 22 | 24 | 23 | 21 | 21 | 24 | 22 | 26 | 23 | 22 |  |  |  |  |  |  |  |
| S. *Lepus yarkandensis* | 6 | 9 | 9 | 3 | 10 | 3 | 9 | 3 | 9 | 7 | 5 | 7 | 10 | 3 | 9 | 3 | 4 | 21 |  |  |  |  |  |  |
| T. *Oryctolagus cuniculus* | 19 | 19 | 21 | 19 | 20 | 20 | 22 | 19 | 23 | 20 | 20 | 16 | 21 | 19 | 23 | 20 | 20 | 23 | 17 |  |  |  |  |  |
| U. *Sylvilagus audubonii* | 16 | 18 | 18 | 14 | 19 | 15 | 18 | 14 | 20 | 15 | 13 | 15 | 19 | 14 | 18 | 15 | 15 | 16 | 14 | 19 |  |  |  |  |
| V. *Sylvilagus brasiliensis* | 12 | 12 | 12 | 12 | 13 | 13 | 17 | 12 | 16 | 13 | 13 | 13 | 18 | 12 | 14 | 13 | 13 | 18 | 14 | 19 | 10 |  |  |  |
| W. *Sylvilagus floridanus* | 16 | 15 | 15 | 16 | 16 | 15 | 18 | 16 | 19 | 17 | 15 | 17 | 19 | 16 | 18 | 15 | 17 | 18 | 16 | 18 | 14 | 8 |  |  |
| X. *Sylvilagus bachmani* | 14 | 14 | 16 | 11 | 15 | 15 | 17 | 11 | 18 | 15 | 15 | 13 | 18 | 11 | 15 | 15 | 12 | 22 | 12 | 19 | 15 | 13 | 16 |  |

**Cyt*b***

|  | A | B | C | D | E | F | G | H | I | J | K | L | M | N | O | P | Q | R | S | T | U | V | W | X | Y | Z | A1 | B1 | C1 | D1 | E1 | F1 | G1 |
| --- | --- | --- | --- | --- | --- | --- | --- | --- | --- | --- | --- | --- | --- | --- | --- | --- | --- | --- | --- | --- | --- | --- | --- | --- | --- | --- | --- | --- | --- | --- | --- | --- | --- |
| A. *Lepus europaeus* |  |  |  |  |  |  |  |  |  |  |  |  |  |  |  |  |  |  |  |  |  |  |  |  |  |  |  |  |  |  |  |  |  |
| B*. Lepus alleni* | 14 |  |  |  |  |  |  |  |  |  |  |  |  |  |  |  |  |  |  |  |  |  |  |  |  |  |  |  |  |  |  |  |  |
| C. *Lepus americanus* | 10 | 10 |  |  |  |  |  |  |  |  |  |  |  |  |  |  |  |  |  |  |  |  |  |  |  |  |  |  |  |  |  |  |  |
| D. *Lepus arcticus* | 11 | 10 | 2 |  |  |  |  |  |  |  |  |  |  |  |  |  |  |  |  |  |  |  |  |  |  |  |  |  |  |  |  |  |  |
| E. *Lepus brachyurus* | 14 | 14 | 9 | 10 |  |  |  |  |  |  |  |  |  |  |  |  |  |  |  |  |  |  |  |  |  |  |  |  |  |  |  |  |  |
| F. *Lepus californicus* | 12 | 5 | 8 | 9 | 12 |  |  |  |  |  |  |  |  |  |  |  |  |  |  |  |  |  |  |  |  |  |  |  |  |  |  |  |  |
| G. *Lepus callotis* | 13 | 5 | 9 | 9 | 13 | 4 |  |  |  |  |  |  |  |  |  |  |  |  |  |  |  |  |  |  |  |  |  |  |  |  |  |  |  |
| H. *Lepus capensis* | 9 | 8 | 3 | 3 | 10 | 7 | 7 |  |  |  |  |  |  |  |  |  |  |  |  |  |  |  |  |  |  |  |  |  |  |  |  |  |  |
| I. *Lepus comus* | 11 | 14 | 9 | 11 | 15 | 12 | 13 | 11 |  |  |  |  |  |  |  |  |  |  |  |  |  |  |  |  |  |  |  |  |  |  |  |  |  |
| J. *Lepus coreanus* | 10 | 11 | 3 | 1 | 9 | 10 | 10 | 4 | 12 |  |  |  |  |  |  |  |  |  |  |  |  |  |  |  |  |  |  |  |  |  |  |  |  |
| K. *Lepus corsicanus* | 12 | 11 | 6 | 6 | 13 | 10 | 10 | 5 | 12 | 7 |  |  |  |  |  |  |  |  |  |  |  |  |  |  |  |  |  |  |  |  |  |  |  |
| L. *Lepus flavigularis* | 11 | 3 | 7 | 7 | 11 | 2 | 2 | 5 | 11 | 8 | 8 |  |  |  |  |  |  |  |  |  |  |  |  |  |  |  |  |  |  |  |  |  |  |
| M. *Lepus granatensis* | 10 | 12 | 7 | 6 | 14 | 11 | 10 | 7 | 12 | 7 | 10 | 9 |  |  |  |  |  |  |  |  |  |  |  |  |  |  |  |  |  |  |  |  |  |
| N. *Lepus hainanus* | 14 | 14 | 13 | 13 | 19 | 11 | 12 | 13 | 13 | 13 | 14 | 11 | 13 |  |  |  |  |  |  |  |  |  |  |  |  |  |  |  |  |  |  |  |  |
| O. *Lepus insularis* | 11 | 4 | 7 | 8 | 11 | 1 | 3 | 6 | 11 | 9 | 9 | 1 | 10 | 12 |  |  |  |  |  |  |  |  |  |  |  |  |  |  |  |  |  |  |  |
| P. *Lepus mandshuricus* | 6 | 9 | 9 | 10 | 13 | 7 | 8 | 7 | 13 | 9 | 8 | 6 | 12 | 11 | 6 |  |  |  |  |  |  |  |  |  |  |  |  |  |  |  |  |  |  |
| Q. *Lepus oiostolus* | 8 | 8 | 7 | 7 | 12 | 7 | 7 | 5 | 8 | 8 | 8 | 5 | 8 | 11 | 6 | 8 |  |  |  |  |  |  |  |  |  |  |  |  |  |  |  |  |  |
| R. *Lepus othus* | 11 | 10 | 2 | 0 | 10 | 9 | 9 | 3 | 11 | 1 | 6 | 7 | 6 | 13 | 8 | 10 | 7 |  |  |  |  |  |  |  |  |  |  |  |  |  |  |  |  |
| S. *Lepus peguensis* | 11 | 11 | 12 | 12 | 17 | 8 | 8 | 10 | 15 | 11 | 13 | 8 | 12 | 5 | 9 | 7 | 9 | 12 |  |  |  |  |  |  |  |  |  |  |  |  |  |  |  |
| T. *Lepus saxatilis* | 11 | 11 | 11 | 11 | 15 | 10 | 9 | 9 | 17 | 12 | 12 | 8 | 11 | 16 | 9 | 10 | 11 | 11 | 13 |  |  |  |  |  |  |  |  |  |  |  |  |  |  |
| U. *Lepus sinensis* | 12 | 15 | 13 | 12 | 17 | 15 | 15 | 10 | 15 | 12 | 13 | 13 | 13 | 15 | 14 | 10 | 13 | 12 | 15 | 13 |  |  |  |  |  |  |  |  |  |  |  |  |  |
| V. *Lepus timidus* | 11 | 10 | 3 | 1 | 10 | 9 | 9 | 2 | 11 | 2 | 5 | 7 | 6 | 13 | 8 | 9 | 7 | 1 | 12 | 11 | 11 |  |  |  |  |  |  |  |  |  |  |  |  |
| W. *Lepus townsendii* | 10 | 9 | 1 | 1 | 9 | 8 | 8 | 2 | 10 | 2 | 5 | 6 | 6 | 12 | 7 | 9 | 6 | 1 | 11 | 10 | 12 | 2 |  |  |  |  |  |  |  |  |  |  |  |
| X. *Lepus yarkandensis* | 12 | 9 | 8 | 7 | 15 | 8 | 8 | 5 | 12 | 8 | 6 | 6 | 8 | 14 | 7 | 8 | 6 | 7 | 11 | 12 | 11 | 6 | 7 |  |  |  |  |  |  |  |  |  |  |
| Y. *Oryctolagus cuniculus* | 16 | 20 | 17 | 17 | 18 | 17 | 19 | 15 | 24 | 17 | 16 | 17 | 17 | 18 | 18 | 16 | 16 | 17 | 16 | 14 | 16 | 17 | 16 | 17 |  |  |  |  |  |  |  |  |  |
| Z. *Sylvilagus aquaticus* | 19 | 22 | 21 | 22 | 18 | 18 | 21 | 20 | 26 | 22 | 25 | 19 | 24 | 22 | 19 | 20 | 21 | 22 | 20 | 18 | 23 | 22 | 21 | 23 | 15 |  |  |  |  |  |  |  |  |
| A1. *Sylvilagus audubonii* | 18 | 19 | 15 | 16 | 16 | 18 | 17 | 16 | 16 | 16 | 17 | 17 | 20 | 21 | 17 | 18 | 16 | 16 | 22 | 21 | 21 | 16 | 15 | 19 | 23 | 21 |  |  |  |  |  |  |  |
| B1. *Sylvilagus brasiliensis* | 14 | 11 | 11 | 12 | 13 | 10 | 11 | 10 | 13 | 13 | 11 | 9 | 16 | 18 | 9 | 11 | 12 | 12 | 17 | 11 | 14 | 12 | 11 | 13 | 19 | 24 | 18 |  |  |  |  |  |  |
| C1. *Sylvilagus floridanus* | 14 | 13 | 13 | 14 | 14 | 13 | 14 | 12 | 18 | 14 | 17 | 12 | 17 | 19 | 12 | 13 | 15 | 14 | 17 | 12 | 15 | 14 | 13 | 16 | 17 | 15 | 17 | 16 |  |  |  |  |  |
| D1. *Sylvilagus nuttallii* | 17 | 21 | 17 | 18 | 17 | 20 | 21 | 16 | 25 | 18 | 18 | 19 | 20 | 24 | 19 | 16 | 19 | 18 | 22 | 16 | 17 | 18 | 17 | 20 | 13 | 19 | 18 | 17 | 15 |  |  |  |  |
| E1. *Sylvilagus obscurus* | 16 | 14 | 12 | 12 | 13 | 11 | 13 | 11 | 18 | 13 | 16 | 11 | 14 | 20 | 10 | 16 | 12 | 12 | 19 | 11 | 18 | 12 | 12 | 13 | 18 | 18 | 17 | 16 | 12 | 19 |  |  |  |
| F1. *Sylvilagus palustris* | 16 | 12 | 13 | 14 | 13 | 14 | 15 | 12 | 19 | 14 | 17 | 13 | 17 | 22 | 13 | 16 | 16 | 14 | 20 | 15 | 19 | 14 | 13 | 17 | 19 | 16 | 18 | 13 | 12 | 16 | 14 |  |  |
| G1*. Sylvilagus transitionalis* | 16 | 14 | 12 | 12 | 13 | 11 | 13 | 11 | 18 | 13 | 16 | 11 | 14 | 20 | 10 | 16 | 12 | 12 | 19 | 11 | 18 | 12 | 12 | 13 | 18 | 18 | 17 | 16 | 12 | 19 | 0 | 14 |  |

**16S rRNA**

|  | A | B | C | D | E | F | G | H | I | J | K | L | M | N | O | P | Q | R |
| --- | --- | --- | --- | --- | --- | --- | --- | --- | --- | --- | --- | --- | --- | --- | --- | --- | --- | --- |
| A. *Lepus europaeus* |  |  |  |  |  |  |  |  |  |  |  |  |  |  |  |  |  |  |
| B. *Lepus americanus* | 11 |  |  |  |  |  |  |  |  |  |  |  |  |  |  |  |  |  |
| C. *Lepus arcticus* | 9 | 10 |  |  |  |  |  |  |  |  |  |  |  |  |  |  |  |  |
| D. *Lepus californicus* | 11 | 8 | 8 |  |  |  |  |  |  |  |  |  |  |  |  |  |  |  |
| E. *Lepus capensis* | 9 | 10 | 2 | 8 |  |  |  |  |  |  |  |  |  |  |  |  |  |  |
| F. *Lepus coreanus* | 9 | 10 | 0 | 8 | 2 |  |  |  |  |  |  |  |  |  |  |  |  |  |
| G. *Lepus granatensis* | 8 | 9 | 1 | 9 | 3 | 1 |  |  |  |  |  |  |  |  |  |  |  |  |
| H. *Lepus hainanus* | 7 | 7 | 10 | 9 | 9 | 10 | 9 |  |  |  |  |  |  |  |  |  |  |  |
| I. *Lepus othus* | 9 | 10 | 0 | 8 | 2 | 0 | 1 | 10 |  |  |  |  |  |  |  |  |  |  |
| J. *Lepus sinensis* | 14 | 11 | 9 | 11 | 9 | 9 | 10 | 12 | 9 |  |  |  |  |  |  |  |  |  |
| K. *Lepus tibetanus* | 10 | 9 | 6 | 9 | 8 | 6 | 5 | 9 | 6 | 11 |  |  |  |  |  |  |  |  |
| L. *Lepus timidus* | 10 | 9 | 1 | 7 | 3 | 1 | 2 | 9 | 1 | 8 | 5 |  |  |  |  |  |  |  |
| M. *Lepus townsendii* | 10 | 9 | 1 | 7 | 1 | 1 | 2 | 9 | 1 | 8 | 7 | 2 |  |  |  |  |  |  |
| N. *Lepus tolai* | 10 | 9 | 3 | 9 | 1 | 3 | 4 | 10 | 3 | 10 | 9 | 4 | 2 |  |  |  |  |  |
| O. *Oryctolagus cuniculus* | 17 | 18 | 21 | 19 | 20 | 21 | 20 | 15 | 21 | 22 | 19 | 20 | 21 | 21 |  |  |  |  |
| P. *Sylvilagus bachmani* | 25 | 24 | 27 | 26 | 26 | 27 | 27 | 22 | 27 | 26 | 25 | 26 | 26 | 27 | 23 |  |  |  |
| Q. *Sylvilagus brasiliensis* | 22 | 21 | 23 | 25 | 23 | 23 | 22 | 21 | 23 | 26 | 20 | 23 | 23 | 24 | 20 | 17 |  |  |
| R. *Sylvilagus floridanus* | 22 | 19 | 24 | 24 | 23 | 24 | 23 | 19 | 24 | 23 | 21 | 23 | 23 | 24 | 22 | 15 | 12 |  |
